# Filling Teeth

**Published:** 1858-12

**Authors:** J. Taft


DENTAL REGISTER OF THE WEST.

VOL. XII.—DECEMBER, 1858.—NO. 2.

### Original Essays and Communications.

### FILLING TEETH.

BY J. TAFT.

### CRYSTAL GOLD.

THE form of the cavity for crystal-gold filling, should be much the same as that described for other fillings, except that the same care is not necessary for special retaining-points, for the first portion of gold that is introduced into the cavity, as good crystal gold, will attach to the wall of the cavity without any such special retaining-points: such a form should be given as to secure the first piece firmly in place. The gold should be cut or broken into pieces corresponding in size to the cavity, so that they will enter freely into it.

The filling may be commenced upon the bottom of the cavity, or upon one of its sides; such a point always being selected as will most effectually retain the gold in place. The pluggers should be of various sizes,—the first one as large as can be used in the cavity, and smaller ones for condensing more thoroughly; and all should be serrated with from two to six sharp points, usually four. The blocks may be taken up on the points of the plugger, and passed to their proper position in the cavity, and there condensed. The sharp, serrated point leaves the surface in good condition for the reception of the next piece. The gold should be packed to the walls of the cavity a little in advance of the center, so that its adaptation may be more complete. In this manner, the filling is built up as much as is desirable, if it is kept dry,—and unless it is, all cohesion is lost.

The gold, after it is cut up, is put on a piece of charcoal, and annealed with a blowpipe, by the flame of a spirit-lamp, carefully, so as not to fuse any of the particles, as that would impair their facility of cohesion in this process: very minute portions are often required to fill up small interstices, or notches.

In crown cavities, the filling should begin at the bottom; in proximal cavities, at the cervical wall. By introducing the gold in this manner, the pressure is made on a line with the axis of the tooth; which is an important consideration. The surface of the filling should always be convex, though in some cases but slightly, except where the antagonism of the teeth prevents, as in the crown fillings of the molars,—in which case, it should be adapted for the reception of the opposing tooth. Proximal fillings should usually be a little convex; yet many good fillings of this class are effected with a surface perfectly plain with the borders of the cavity. The borders of the filling, however, are better protected when it is somewhat convex.

Crystal gold, of perfect character, presents to the walls of the cavity a surface better calculated to be retained, than foil in any of its forms; though adhesive foil possesses this advantage to a greater extent than foil in any other condition. The points and edges of the crystals are brought in contact with the walls, and made to take a firmer hold upon the dentine.

In forming crystal gold into a solid mass, two principles are operative: cohesion acts upon it as potentially as upon gold in any other form, and, in addition, there is the interlacing, or locking, of the crystals with one another; so that a more perfect union of the different portions of which a filling is composed, is obtained with crystal gold than with foil. Yet, good adhesive foil, when thoroughly worked in, attains almost the same condition. With perfect crystal gold, however, some advantages may be secured, that can not be had with foil. The gold will be retained in a cavity that will not retain a foil filling; it is more easily formed into a coherent mass; it can be built out with more ease; cavities can be filled with it that can not be filled with foil at all; and a filling of it is susceptible of a far better finish, with the same labor, than a filling of foil.

It is important to keep the gold perfectly free from moisture, while being introduced and consolidated; for moisture instantly destroys the cohesive property of the gold. And the more complete the exclusion of moisture from the cavity, during the process, the better for the success of the operation. The surface of every filling should be consolidated for finishing, before it is allowed to become moist; for, whenever it becomes saturated with moisture before consolidation, it is impossible to make a perfect finish. There should always be gold enough superadded to insure this; and the consolidation of the last surface should be effected with a rough-ended, not serrated, instrument.

*Finishing Fillings.*—The method of finishing a filling, and the manipulation, will depend somewhat on the locality of the cavity. When this has been completely filled, and thoroughly consolidated over all the surface, and especially all round the border of the plug, the file should be applied to dress off any projecting portion, and render the filling smooth. In consolidating the surface, an instrument should be used, that would not pit it, and the file should remove all indentations. The work of the file, however, should be but partially performed at first, and the surface consolidated again, with a square-pointed instrument. To obtain the most perfect finish, the surface should be brought to a uniform consistence; and this condition can not be reached by the use of sharp-pointed instruments, nor fully by that of the blunt plugger at the first effort, but by the alternate use of the file, the blunt condenser, and the burnisher. A coarse file should be employed in this part of the process; but when the filling is dressed sufficiently, and in good condition, the fine file should be used, alternately with the burnisher, till a perfectly uniform surface is obtained. In all cases, after the file has been applied, the plug should be washed off with a brush, to remove all detached pieces of gold, before the burnisher is put upon it; and after the fine file and burnisher, the Scotch or Arkansas stone or very fine pumice should be employed to remove the file-marks. The pumice may be applied with water on a strip of chamois skin, a piece of linen tape, or a stick of soft wood,—the latter being the most convenient, as it can be used with one hand and shaped to suit any place or position.

After the stone or the pumice has accomplished its work, and the filling has been thoroughly washed, a fine burnisher, with a solution of castile-soap, is employed to give the finish. The burnisher should be of the best cast steel, of high temper and fine polish. Considerable skill is requisite to give the best effect with the burnisher; it should pass smoothly and gently over the surface, throughout its whole extent, and in parallel lines, with a pressure neither too light nor too heavy. It should also be applied very thoroughly upon any portion of the tooth about the border of the filling, that may have been cut by the file or any other instrument. Indeed, quite as much—if not more—care should be exercised upon this as upon the plug itself: it should be polished as smooth as the enamel, if possible; for the more nearly perfect it is in this respect, the better will it resist the action of deleterious agents.

This method of finishing gives to the filling a perfect metallic luster; which, under some circumstances, might be objectionable. Two other methods are in use: After the burnisher has been applied, as above, the buff or tape, with rouge, may be employed, by passing it rapidly over the filling till the metallic luster is destroyed, or deadened so as not to reflect the light as before,—thus leaving a very desirable finish.

The other method is, to stipple over the surface of the burnished filling with the end of a piece of hard wood,—sandal wood is recommended,—charged with finely pulverized pumice. This gives a beautiful, velvet-like surface, and is fine for fillings in the anterior portion of the mouth, where they are exposed to view. Rotten stone, applied either with the buff or with hard wood, imparts a finish which, though a little different, is finer than any of the others.

For finishing, some operators prefer to cut and polish, instead of filing and burnishing. But neither so good nor so fine a finish can be effected in this way, and it is probable that economy of time and labor, especially the latter, suggested the method.

In all cases, the filling should have a distinct and difinite margin: the gold should be trimmed off quite up to the border of the cavity, by passing round it a small, sharp instrument, so as to detect and pare down any portion that might overlay the tooth. For, if overlapping portions are permitted to remain, foreign substances will lodge beneath, and induce decay. Neglect in this particular has occasioned the loss of thousands of teeth that otherwise might have been saved.

The subject of finishing is almost entirely overlooked by very many operators; but by the neat and skillful it is esteemed of sufficient importance to demand as great labor and pains as any other part of the operation.

